# Supplementary material for: Nitrogen uptake preference of cotton (Gossypium hirsutum L.)
Source: PLoS One. 2025 Dec 12;20(12):e0334700. doi: 10.1371/journal.pone.0334700 (PMC12700366; doi:10.1371/journal.pone.0334700)
Supplement: S1 Fig — (A) Injection of rhizotube with N solution. Careful attention was paid to not inject solution directly into the plant. (B) Extracted and cleaned G. hirsutum seedling. Once cleaned, seedlings were bagged and placed on dry ice (CO2(s)) to halt metabolism. (DOCX) [file pone.0334700.s001.docx]

Supplement Figures

| 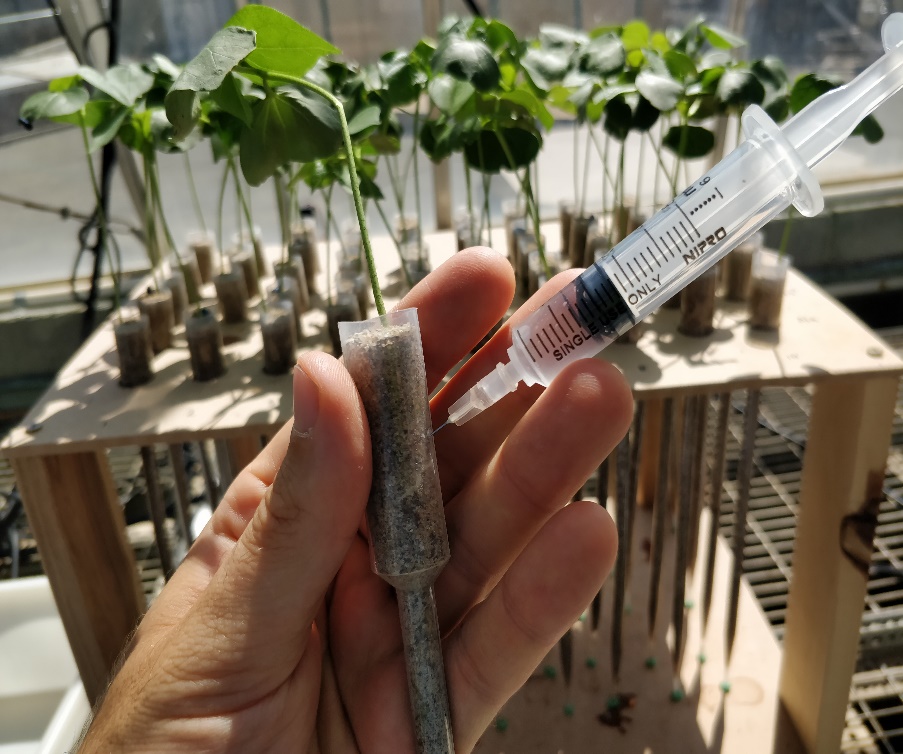  **(A)**  **(B)** | 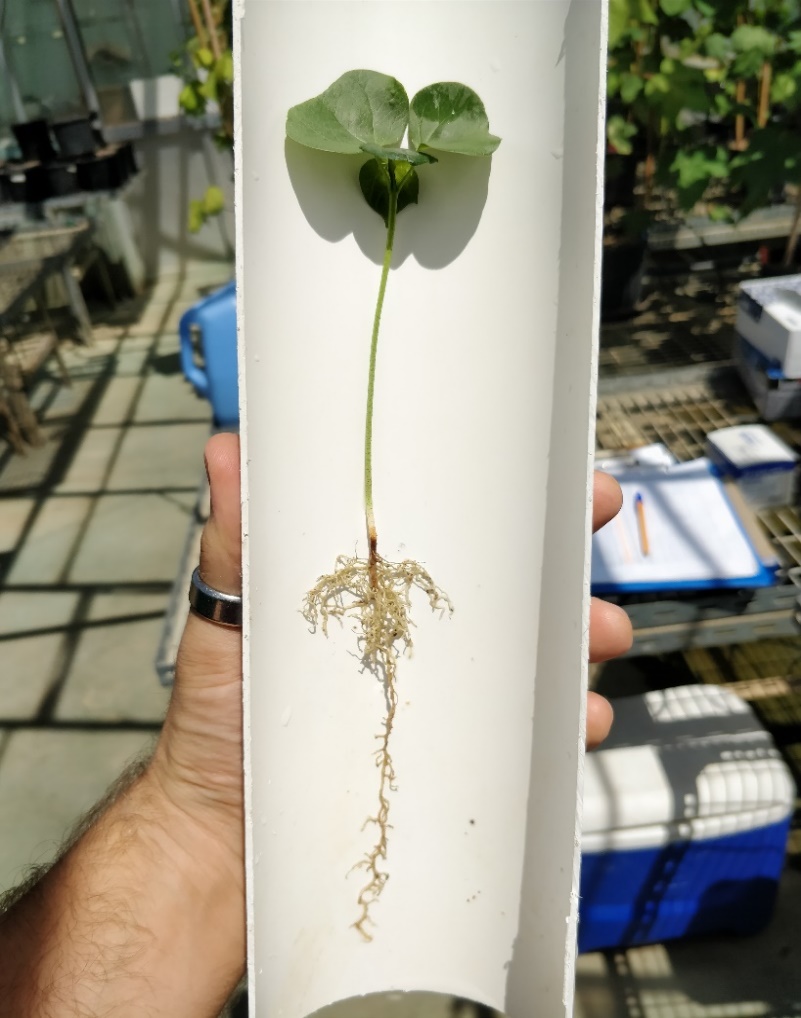 |
| --- | --- |
|  |  |

**Figure S1.** Processing of *G. hirsutum* seedlings in ^15^N^13^C uptake glasshouse experiment. **(A)** Injection of rhizotube with N solution. Careful attention was paid to not inject solution directly into the plant. **(B)** Extracted and cleaned *G. hirsutum* seedling. Once cleaned, seedlings were bagged and placed on dry ice (CO_2(s)_) to halt metabolism.
