# Supplementary material for: Nitrogen uptake preference of cotton (Gossypium hirsutum L.)
Source: PLoS One. 2025 Dec 12;20(12):e0334700. doi: 10.1371/journal.pone.0334700 (PMC12700366; doi:10.1371/journal.pone.0334700)
Supplement: S2 Fig — n = 720 seeds. (DOCX) [file pone.0334700.s002.docx]

Figure S2

|  |
| --- |
| **Figure S2.** Germination percentage for each of the three G. hirsutum varieties: Sicot 746B3F, a GM current commercial cultivar; Sicala V2, an obsolete non-GM commercial cultivar; and Tx III, a Guatemalan landrace accession. n = 720 seeds. |
