# Supplementary material for: Nitrogen uptake preference of cotton (Gossypium hirsutum L.)
Source: PLoS One. 2025 Dec 12;20(12):e0334700. doi: 10.1371/journal.pone.0334700 (PMC12700366; doi:10.1371/journal.pone.0334700)
Supplement: Attachment — Submitted filename: PONE-D-24-59331_edited.pdf [file pone.0334700.s004.pdf]

PLOS ONE

Nitrogen uptake preference of Gossypium hirsutum L. from a mixture of urea, alanine, nitrate, and ammonium

--Manuscript Draft--

**Manuscript Number:**	PONE-D-24-59331
**Article Type:**	Research Article
**Full Title:**	Nitrogen uptake preference of Gossypium hirsutum L. from a mixture of urea, alanine, nitrate, and ammonium
**Short Title:**	Nitrogen uptake capability in cotton
**Corresponding Author:**	Mark Farrell, PhD<br>Commonwealth Scientific and Industrial Research Organisation<br>Glen Osmond, SA AUSTRALIA
**Keywords:**	Dissolved organic nitrogen; crop nutrition; nitrogen cycling; irrigated cotton
**Abstract:**	Low molecular weight (LMW) soil organic nitrogen (N) can be a significant source of N in commercial cotton (Gossypium hirsutum L.) systems, potentially comprising a meaningful portion of N uptake in Australian irrigated cotton. Cotton obtains the majority of its N from the soil N pool rather than directly from fertiliser-N, with organic N being a major component of this. We tested the ability of G. hirstutum to take up different organic and inorganic N forms in a sand matrix, greatly reducing potential for microbial competition and enabling a clearer view of the physiological capacity the plant to access different N forms. The experiment showed that cotton took up inorganic N (NO3- and NH4+) and organic N (alanine and urea) concurrently, with a slight preference for inorganic N overall. The uptake mechanism for organic carbon (C) associated with the organic N was also examined, showing that alanine-C was taken up linearly, with a consistent internal 13C:15N ratio suggesting that some alanine was absorbed intact. This study expands the list of plants known to utilise organic N to include commercial cotton. Overall, the experiment demonstrated that G. hirsutum can rapidly access different soil N pools, with a slight preference for inorganic N, and that the uptake mechanisms for organic N and C are complex and warrant further investigation.
**Order of Authors:**	James O Latimer, PhD
	Mark Farrell, PhD
	Ben CT Macdonald, PhD
**Additional Information:**	
**Question**	**Response**
**Financial Disclosure**<br><br>Enter a financial disclosure statement that describes the sources of funding for the work included in this submission. Review the submission guidelines for detailed requirements. View published research articles from *PLOS ONE* for specific examples.<br><br>This statement is required for submission and **will appear in the published article** if the submission is accepted. Please make sure it is accurate.	Yes

Powered by Editorial Manager® and ProduXion Manager® from Aries Systems Corporation

**Funded studies**<br>Enter a statement with the following details:<br>• Initials of the authors who received each award<br>• Grant numbers awarded to each author<br>• The full name of each funder<br>• URL of each funder website<br>• Did the sponsors or funders play any role in the study design, data collection and analysis, decision to publish, or preparation of the manuscript?<br><br>Did you receive funding for this work?	
Please add funding details.<br>as follow-up to "**Financial Disclosure**<br><br>Enter a financial disclosure statement that describes the sources of funding for the work included in this submission. Review the submission guidelines for detailed requirements. View published research articles from *PLOS ONE* for specific examples.<br><br>This statement is required for submission and **will appear in the published article** if the submission is accepted. Please make sure it is accurate.<br><br>**Funded studies**<br>Enter a statement with the following details:<br>• Initials of the authors who received each award<br>• Grant numbers awarded to each author<br>• The full name of each funder<br>• URL of each funder website<br>• Did the sponsors or funders play any role in the study design, data collection and analysis, decision to publish, or preparation of the manuscript?<br><br>Did you receive funding for this work?"	The authors gratefully acknowledge the Cotton Research and Development Corporation (https://crdc.com.au/; Grant number CSP1904 awarded to BM and MF) for funding. The funders played no role in the study design, data collection and analysis, decision to publish, or preparation of the manuscript.
Please select the country of your main research funder (please select carefully as in some cases this is used in fee	AUSTRALIA - AU

calculation).<br>as follow-up to "**Financial Disclosure**<br><br>Enter a financial disclosure statement that describes the sources of funding for the work included in this submission. Review the submission guidelines for detailed requirements. View published research articles from *PLOS ONE* for specific examples.<br><br>This statement is required for submission and **will appear in the published article** if the submission is accepted. Please make sure it is accurate.<br><br>**Funded studies**<br>Enter a statement with the following details:<br>• Initials of the authors who received each award<br>• Grant numbers awarded to each author<br>• The full name of each funder<br>• URL of each funder website<br>• Did the sponsors or funders play any role in the study design, data collection and analysis, decision to publish, or preparation of the manuscript?<br><br>Did you receive funding for this work?"	
**Competing Interests**<br><br>Use the instructions below to enter a competing interest statement for this submission. On behalf of all authors, disclose any competing interests that could be perceived to bias this work—acknowledging all financial support and any other relevant financial or non-financial competing interests.<br><br>This statement is required for submission and **will appear in the published article** if the submission is accepted. Please make sure it is accurate and that any funding sources listed in your Funding Information later in the submission form are also declared in your Financial Disclosure	he authors have declared that no competing interests exist.

statement.<br><br>View published research articles from *PLOS ONE* for specific examples.<br><br>**NO authors have competing interests**<br><br>Enter: *The authors have declared that no competing interests exist.*<br><br>**Authors with competing interests**<br><br>Enter competing interest details beginning with this statement:<br><br>*I have read the journal's policy and the authors of this manuscript have the following competing interests: [insert competing interests here]*<br><br>* typeset	
**Ethics Statement**<br><br>Enter an ethics statement for this submission. This statement is required if the study involved:<br><br>• Human participants<br>• Human specimens or tissue<br>• Vertebrate animals or cephalopods<br>• Vertebrate embryos or tissues<br>• Field research<br><br>Write "N/A" if the submission does not require an ethics statement.<br><br>General guidance is provided below. Consult the submission guidelines for detailed instructions. **Make sure that all information entered here is included in the Methods section of the manuscript.**	N/A

**Format for specific study types**<br><br>**Human Subject Research (involving human participants and/or tissue)**<br>• Give the name of the institutional review board or ethics committee that approved the study<br>• Include the approval number and/or a statement indicating approval of this research<br>• Indicate the form of consent obtained (written/oral) or the reason that consent was not obtained (e.g. the data were analyzed anonymously)<br><br>**Animal Research (involving vertebrate animals, embryos or tissues)**<br>• Provide the name of the Institutional Animal Care and Use Committee (IACUC) or other relevant ethics board that reviewed the study protocol, and indicate whether they approved this research or granted a formal waiver of ethical approval<br>• Include an approval number if one was obtained<br>• If the study involved *non-human primates*, add *additional details* about animal welfare and steps taken to ameliorate suffering<br>• If anesthesia, euthanasia, or any kind of animal sacrifice is part of the study, include briefly which substances and/or methods were applied<br><br>**Field Research**<br><br>Include the following details if this study involves the collection of plant, animal, or other materials from a natural setting:<br>• Field permit number<br>• Name of the institution or relevant body that granted permission	
**Data Availability**<br><br>Authors are required to make all data underlying the findings described fully available, without restriction, and from the time of publication. PLOS allows rare exceptions to address legal and ethical concerns. See the PLOS Data Policy and FAQ for detailed information.	Yes - all data are fully available without restriction

<table>
<tr>
<td>A Data Availability Statement describing where the data can be found is required at submission. Your answers to this question constitute the Data Availability Statement and will be published in the article, if accepted.

Important: Stating 'data available on request from the author' is not sufficient. If your data are only available upon request, select 'No' for the first question and explain your exceptional situation in the text box.

Do the authors confirm that all data underlying the findings described in their manuscript are fully available without restriction?</td>
<td></td>
</tr>
<tr>
<td>Describe where the data may be found in full sentences. If you are copying our sample text, replace any instances of XXX with the appropriate details.

• If the data are held or will be held in a public repository, include URLs, accession numbers or DOIs. If this information will only be available after acceptance, indicate this by ticking the box below. For example: All XXX files are available from the XXX database (accession number(s) XXX, XXX.).
• If the data are all contained within the manuscript and/or Supporting Information files, enter the following: All relevant data are within the manuscript and its Supporting Information files.
• If neither of these applies but you are able to provide details of access elsewhere, with or without limitations, please do so. For example:

Data cannot be shared publicly because of [XXX]. Data are available from the XXX Institutional Data Access / Ethics Committee (contact via XXX) for researchers who meet the criteria for access to confidential data.

The data underlying the results presented in the study are available from (include the name of the third party</td>
<td>All relevant data will be held and made publicly within the CSIRO Data Access Portal (https://data.csiro.au). A unique DOI will be generated upon publication.</td>
</tr>
</table>

and contact information or URL).<br>• This text is appropriate if the data are owned by a third party and authors do not have permission to share the data.<br><br>* typeset	
Additional data availability information:	Tick here if the URLs/accession numbers/DOIs will be available only after acceptance of the manuscript for publication so that we can ensure their inclusion before publication.

Powered by Editorial Manager® and ProduXion Manager® from Aries Systems Corporation

Manuscript Click here to access/download;Manuscript;latimeretal_v4.docx

Nitrogen uptake preference of *Gossypium hirsutum* L. from a mixture of urea, alanine, nitrate, and ammonium

Latimer, J.O.[1, 2 *]; Farrell, M.[2*]; Macdonald, B.C.T.[2]

[1]The Fenner School of Environment and Society, the Australian National University, Canberra ACT

[2]Commonwealth Scientific and Industrial Research Organisation

*Corresponding authors:

J.O. Latimer: The Australian National University, Fenner School of Environment and Society, 141 Linnaeus Way, Acton ACT 2601, Australia. Email Address: James.Latimer@anu.edu.au (Latimer, J.O.)

M. Farrell: Commonwealth Scientific and Industrial Research Organisation, PO Box 200, Glenside, SA 5251, Australia: Email address: mark.farrell@csiro.au (Farrell, M.)

Short title: Nitrogen uptake capability in cotton

Abstract

Low molecular weight (LMW) soil organic nitrogen (N) can be a significant source of N in commercial cotton (*Gossypium hirsutum* L.) systems, potentially comprising a meaningful portion of N uptake in Australian irrigated cotton. Cotton obtains the majority of its N from the soil N pool rather than directly from fertiliser-N, with organic N being a major component of this. We tested the ability of *G. hirstutum* to take up different organic and inorganic N forms in a sand matrix, greatly reducing potential for microbial competition and enabling a clearer view of the physiological capacity the plant to access different N forms. The experiment showed that cotton took up inorganic N (NO_3^- and NH_4^+) and organic N (alanine and urea) concurrently, with a slight preference for inorganic N overall. The uptake mechanism for organic carbon (C) associated with the organic N was also examined, showing that alanine-C was taken up linearly, with a consistent internal ^{13}C:^{15}N ratio suggesting that some alanine was absorbed intact. This study expands the list of plants known to utilise organic N to include commercial cotton. Overall, the experiment demonstrated that *G. hirsutum* can rapidly access different soil N pools, with a slight preference for inorganic N, and that the uptake mechanisms for organic N and C are complex and warrant further investigation.

1. Introduction

The cotton fibre produced from *Gossypium hirsutum L.* farming systems is globally the most widely utilised natural fibre (1). It was estimated by Heffer and Prud'homme (2) in 2014 that the global fibre crop, which is mostly cotton, uses 4.3% of the global production of nitrogen (N) fertiliser (110.4 Tg N) to produce 25.9 Tg of lint (3). The global N use efficiency index is 6 kg lint kg^{-1} applied N which is outside the recommend optimum range of 13-18 kg lint kg^{-1} applied N (4, 5). This indicates that the global crop is potentially receiving more fertiliser N than required, and may be subsequently impacting environmental processes across the globe (6). This apparent over fertilsation results in N run-off losses (7), deep drainage (8) and ground water pollution (9), and greenhouse gas production (10). Brackin et al. (11) showed that despite the significant amounts of applied N fertiliser, cotton crop fertiliser recovery is only between 27–38%. This indicates that a substantial proportion of crop N is derived from the antecedent soil N pool. It was assumed by Brackin et al. (11) that cotton N uptake is from the inorganic pool after minerialisation despite the presence of organic N pool within most soils.

Soil organic N is derived from soil organic matter (SOM), which typically constitutes the largest soil N pool, and contains more C than the atmosphere and global vegetation combined (12, 13). Globally, soils have lost approximately half of their C stocks since the adoption of soil cultivation techniques (14) and therefore also potentially 50% of their N associated with the C in SOM. Soil organic matter is frequently undervalued when considering crop nutrition, with the focus often directed at inorganic N (NO_3^- and NH_4^+) sources from fertiliser. To simplify N management, N-bearing molecules are commonly aggregated into different pools based on context-specific functionality, which typically are: inorganic N, dissolved organic N (DON), synthetic N, and insoluble organic N pools. The size of these pools can vary considerably throughout the year (15) because N continually fluxes between these different compounds, with no molecule providing permanent immobilisation (12).

Since the mid-19th century and the dismissal of the humus theory of plant nutrition, the dominant agricultural paradigm has held that plants only take up N from the soil in the inorganic forms of NH_4^+ and NO_3^-. This limited view of plant nutrition pervaded to the end of the 20th century, likely due to

the ease with which inorganic N can be measured, the fact that it is definitely plant-available, and an increased understanding of the role microbes play in soil N cycling combined with an over-extrapolation of plants' reliance on them for nutrition (16). In addition, the rise of Haber-Bosch-derived fertilisers at the beginning of the 20th century shifted the focus away from organic soil amendments and fixation as the primary means of increasing plant-available N. With productivity gains so easily achieved by synthetic fertilisers, the research community and industry shifted its focus towards NH_4^+ and NO_3^- and maximising yield.

Research demonstrating the importance of organic N in plant nutrition is not new (17-19). However, it was not until the discoveries that organic soil N can be the principal N pool for some plants (20), and that low molecular weight (LMW) DON molecules can be taken up intact by plants (21, 22) that the mainstream view of terrestrial N dynamics shifted to include direct plant uptake of DON from the soil. The uptake of DON has been documented in many plant species, but has not yet been observed in commercial cotton (*G. hirsutum*). No previous experiments have demonstrated *G. hirsutum*'s ability to take up organic N, nor its preference for one N species over another.

This paper reports on four experimental hypotheses. The first was that *G. hirsutum* would not exhibit a N uptake preference between the different N species (NO_3^-, NH_4^+, urea and alanine). The second hypothesis was that alanine—N and alanine—C uptake would correlate, indicating that alanine is taken up whole by *G. hirsutum*. The third hypothesis was that urea—N and urea—C uptake would correlate, indicating that urea is taken up whole by *G. hirsutum*. The fourth experimental hypothesis was that the three *G. hirsutum* varieties (transgenic, non GM, original landrace) would not exhibit different N uptake behaviours. This experiment was not designed to emulate natural conditions, but rather to assess the plant's physiological capabilities and preferences.

2. Materials and Methods

2.1 Experimental Design

Three varieties of *G. hirsutum* were grown to a two- to four-leaf stage in a randomised block design with 10 replicates. The three varieties used were: Sicot 746B3F, a GM current commercial cultivar accounting for more than half (54%) of the 2017-18 Australian summer plantings; Sicala V2, an obsolete non-GM commercial cultivar released in 1994; and Tx III, a Guatemalan landrace accession that represents the native origins of the commercial *G. hirsutum* varieties. Plants were grown in 300 mm deep low-density polyethylene (LDPE) pots, consistent with the rhizotube design used by Hill and Jones (23). Tubes were open-bottomed, allowing drainage and preventing waterlogging. Pasteurised and washed sand of <1 mm particle size was used as the growing medium to enable control over plant-available N pools and to limit the potential of microbial competition for the applied N compounds. Plants were watered using a nutrient solution mixture that constituted 100% of the plants' soil-derived nutrient supply (Table 1). The plants were grown in glasshouses at the CSIRO Black Mountain site in Canberra, ACT, Australia (35 °16'S 149 °7'E) in January (summer) 2018. The high solar radiation experienced by Canberra in summer required the use of 70% shade cloth, as sand temperatures exceeded 60°C when exposed to direct sunlight. In total, 450 seedlings were analysed, comprising 10 replicates each of 45 unique treatments: five isotopically-labelled N and control treatments, three *G. hirsutum* varieties, and four sampling times (0, 5, 60 and 180 minutes).

2.2 Plant Labelling and Processing

All plants were dosed with a N solution containing a mixture of NO_3^-, NH_4^+, urea and alanine. One of the four N compounds was isotopically-labelled in each treatment, with a fifth control treatment containing no labelling (Table 2). The four N compounds were supplied in the same concentration to every seedling, proportionally to the three bioavailable N pools found in intensive agricultural environments: ~33% inorganic N (NO_3^- and NH_4^+), ~33% DON (alanine), and ~33% urea N (urea).

Once plants reached a two- to four-leaf stage, the rhizotube pots were injected with 2 ml of solution at three points around the root base, with special care taken to avoid injecting into any roots (Fig.

S1). Plants were then placed in direct sunlight for their allocated uptake period (5, 60 and 180 minutes). After the treatment times had elapsed, plants were carefully extracted from their pots, thoroughly washed of substrate, bagged, and placed on dry ice ($CO_{2\,(s)}$) to halt metabolism. Plants were transferred to ovens to air dry (at 40°C) completely at the end of each day. Dried samples were weighed and ground to a fine powder using a ball-mill. Ground samples were analysed for $\delta^{13}C$ and $\delta^{15}N$ using isotope ratio mass spectrometry (IRMS) at the Research School of Biology at the ANU, Canberra, ACT. Total C and N were measure on an Elementar VarioMAX CNS elemental analyser.

2.3 Caluculation of ^{13}C and ^{15}N uptake

The process of transposing relative $\delta^{15}N$ and $\delta^{13}C$ values to absolute percentage uptake values followed Equations 1-4. All equations listed below were used for both N and C calculations, with the ^{15}N and ^{14}N equation pronumerals substituted with ^{13}C and ^{12}C respectively in the C calculations.

The calculation of mass fraction (X) from relative isotopic enrichment (δ). $R(^{13}C)_{standard} = 0.011178$ and $R(^{15}N)_{standard} = 0.00367647$ followed equation 1

$$X(^{15}N)_{sample} = \frac{R(^{15}N)_{standard} \cdot \left(\frac{\delta^{15}N}{1000} + 1\right)}{1 + \left(R(^{15}N)_{standard} \cdot \left(\frac{\delta^{15}N}{1000} + 1\right)\right)}$$ **Equation 1.**

The calculation of percentage N derived from transfer (%NDFT) from mass fractions (X) of sample, isotope label, and cultivar variety control. $X(^{15}N)_{label} = \%^{15}N\ Purity_{label}$ and $X(^{15}N)_{variety\ control} = (\sum X(^{15}N)_{control}) \div n$, where n = number of replicates (always 10 in this experiment).

$$\%NDFT = \frac{\%\ ^{15}N\ Atom\ Excess_{sample}}{\%\ ^{15}N\ Atom\ Excess_{donor}} \times 100\% = \frac{X(^{15}N)_{sample} - X(^{15}N)_{variety\ control}}{X(^{15}N)_{label} - X(^{15}N)_{variety\ control}} \times 100\%$$ **Equation 2.**

The calculation of the mass of fertiliser ^{15}N taken up by the plant using %NDFT, plant dry mass (grams), and plant percentage N.

$$m(Fert.\ ^{15}N)_{taken\ up} = \%NDFT \times m(Plant)_{dry} \times Plant\ \%N$$ **Equation 3.**

The calculation of percentage fertiliser ^{15}N uptake by the plant using molar mass (M_r) of ^{15}N (15 mg mmol^{-1}), percentage purity (%Purity) of isotope labels (98.0-99.8%), N concentration (mmol L^{-1}), and volume (L).

$$\%\ \text{Fert.}^{15}\text{N Uptake} = \frac{\text{m}(\text{Fert.}^{15}\text{N})_{\text{taken up}}}{\text{m}(\text{Fert.}^{15}\text{N})_{\text{added}}} = \frac{\text{m}(\text{Fert.}\,^{15}\text{N})_{\text{taken up}}}{[\text{Label}-\text{N}]\times\%\text{Purity}\times\text{V}_{fert.sol.}\times\text{M}_\text{r}(^{15}\text{N})} \qquad \textbf{Equation 4.}$$

The calculation of the fraction of the plant uptake ^{15}N to the initial solution solution ^{15}N was determine using the 15N content in the plant and the initial solution.

$$\text{Fraction of initial} = \frac{\text{Fertiliser }^{15}\text{N uptake}}{^{15}\text{N Intital solution}} \qquad \textbf{Equation 5}$$

2.4 – Statisical Analysis

The statistical analyses were performed in R (24) using the lme4 linear mixed effects modelling package (25) was used to determine the preference of N in the different varieties.

3. Results

3.1 Growing phase

The washed and pasteurised sand used as the growing medium total N (0.003% ± 0.0001) and total C (0.029% ± 0.001) concentrations were negligible prior to planting. The three *G. hirsutum* varieties did not exhibit uniform germination characteristics (Fig S2). However, by the date of labelling, the Sicot, Sicala and Tx varieties achieved germination rates of 88%, 97% and 86% respectively.

3.2 Nitrogen compound uptake

All three *G. hirsutum* varieties rapidly utilised the added NO_3^-, NH_4^+, urea (CH_4N_2O) and alanine ($C_3H_7NO_2$; Fig. 1). After 180 minutes, the average plant uptake for each N species was between 14.3 ± 1.9% and 25.1 ± 1.9% of added fertiliser N (Fig. 2; Table 2). All three *G. hirsutum* varieties showed a small preference for inorganic N (NO_3^- and NH_4^+) over organic N (alanine and urea), with respective mean uptakes of added N 20.9 ± 0.04% and 18.3 ± 0.04% for the two N pools (Table 2).

3.3 Timing of nitrogen uptake

Uptake of all N species presented as linear over the 180 minute experiment window, suggesting zero order kinetics over this timeframe. Supplied N was taken up at rates of 4.6-8.1% of added fertiliser N per hour, equating to 0.28-2.11 mg N $hour^{-1}$ (Table 3).

3.4 Cotton variety

Neither total (Fig. 1B) nor N compound uptake (Fig. 2) were statistically different between the three *G. hirsutum* varieties ($p = 0.20$ and $p = 0.47$ respectively). However, differences in average N uptake from the different N compounds (Fig. 6A) were significant ($p < 0.001$). After 180 minutes, the uptake of NH_4^+–N and alanine—N were not statistically different ($p = 0.77$). Neither were NH_4^+–N and urea-N ($p = 0.11$).

The uptake of alanine—N and urea—N represented the only N compound uptake difference within each varieties and were statistically different in the landrace accession Tx III ($p < 0.05$) but not the two commercial cultivars ($p = 0.58$). Uptake of NO_3^-–N and alanine—N, and NO_3^-–N and NH_4^+–N were both statistically different across all varieties ($p < 0.01$), while NO_3^-–N and urea—N were not ($p = 0.63$).

3.5 Nitrogen and carbon uptake

Alanine—C was taken up linearly over the 180 minute window by all *G. hirsutum* varieties, similarly to alanine—N (Fig. 3). There were no statistically significant differences in alanine—C or alanine—N uptake between the three *G. hirsutum* varieties ($p = 0.73$). C and N were taken up in a consistent ratio of approximately 0.32:1 (Fig. 4).

The uptake of urea—C is does not have a linear take-up over the 180 minute experiment window (Fig. 5). Over the full 180 minutes, no correlation was found between urea—C uptake and variety or urea—N uptake ($p = 0.96$; Fig. 6). Internal plant urea—C concentration rose over the first 60 minutes, and then fell back to almost zero by the end of the 180 minutes.

4. Discussion

4.1 Direct Plant Access of Soil Organic Nitrogen

Our results demomnstrate that LMW DON can be a direct source of nutrition for commercial cotton (*G. hirsutum*). In some situations, this may comprise a meaningful portion of Australian irrigated cotton's N uptake. Recent studies have shown that cotton obtains the majority of its N from the soil N pool (26) and that organic N is a major soil N pool in most agricultural soils (15, 27). However, as this experiment was not designed to emulate real-world conditions, further soil-based glasshouse and *in situ* experiments will be required determine if this is the case in more complex non-sterile systems.

G. hirsutum does not appear to exhibit any uptake preferences between the four N species added. All four N species were taken up concurrently, suggesting that cotton is not selective about the form in which it receives N. All three *G. hirsutum* varieties exhibited a small preference for inorganic N (NO_3^- and NH_4^+) over organic N (alanine and urea), with respective mean total uptake of the two N pools 20.9 ± 0.04% and 18.3 ± 0.04% of added N. These research findings are consistent with those observed in Australian sugarcane (*Saccharum officinarum* L.), which is also produced in high-input systems that are comparable to Australian irrigated cotton. Sugarcane has been shown to readily take up soil amino acids, which can constitute a significant source of N for commercial crops (28, 29).

The *G. hirsutum* plants used in this experiment were grown from seed in washed and pasteurised sand. While this may not have completely eliminated mycorrhizal fungi or other soil microorganisms from the growing environment, it will most likely have significantly diminished their presence and role in plant N uptake. This experiment did not test for mycorrhizal activity, but it is a credible assumption that the *G. hirsutum* seedlings took up the majority of the alanine—N and urea—N without the aid of symbiotic mycorrhizal fungi (20, 30, 31).

In recent years, studies have shown that a wide variety of plants can take up organic N, including: trees (32-39), grasses and sedges (28, 29, 40-42), mosses and lichens (41, 43, 44), fruits (45, 46), and broadacre crops including wheat, maize, chicory, and lupine (47-49). This list of plants known to take

up organic N can now be expanded to include commercial cotton (*G. hirsutum*), a high-value, high-input pillar crop.

4.2 Organic Carbon Uptake Mechanism

Similarly to alanine—N, alanine—C was taken up linearly over the 180 minute window by all *G. hirsutum* varieties. The consistent internal seedling ^{13}C:^{15}N ratio of 0.32:1 suggests that approximately 32% of the alanine—N taken up was in the form of whole alanine molecules. Many other studies have already demonstrated the intact uptake of the LMW DON molecules alanine (23), arginine (37), glycine (50), acetate (47) and trialanine (51), as well as plant consumption of whole proteins and microbes (31, 52, 53), but never before in *G. hirsutum*. While it is possible that the alanine—C taken up was not in the form of intact alanine, functionally this is indistinct from intact alanine uptake (Fig. 7). The majority of alanine—N taken up was not associated with alanine—C, suggesting that extracellular deamination is the dominant alanine—N uptake pathway. It is also possible that all absorbed alanine—N was as whole alanine molecules, and that approximately 68% of the alanine—C was subsequently expelled post-intracellular deamination. Regardless, these results suggest that at least some alanine was taken up whole.

In the early stages of growth, much N taken up from the soil is typically transported to the leaves to create proteins like Ribulose-1,5-bisphosphate carboxylase/oxygenase (Rubisco; (54, 55)). Taking up intact amino acids and transporting them whole may provide a biochemical energy advantage over taking up inorganic N and assembling amino acids from it. This should be explored further, with previous studies already confirming that high organic N uptake can alter plant internal N distribution (29).

Urea—C uptake did not follow the same linear trend observed in the other N and C species, with internal plant urea—C concentration increasing over the first 60 minutes and subsequently falling to almost zero by 180 minutes. This was true for all three *G. hirsutum* varieties, with no significant differences between them ($p = 0.34$). One possible mechanistic explanation for this behaviour would be the intact uptake of some urea, followed by intracellular ammonification and subsequent

expulsion of the urea—C as CO_2. While the dominant urea—N uptake pathway appears to involve extracellular ammonification, there is likely additional complexity and this should be explored further in future experiments.

5. Conclusions

This experiment has shown that *G. hirsutum* can rapidly and concurrently access the DON, inorganic N and synthetic N pools in soil. While all N species were taken up, total N uptake from each N compound was different. *G. hirsutum* exhibited a small preference for inorganic N (NO_3^- and NH_4^+) over organic N (alanine and urea), with respective mean uptake of 20.9 ± 0.04% and 18.3 ± 0.04% of added N for the two pools. Urea—N and NO_3^-—N were taken up more than alanine—N and NH_4^+—N. The consistent uptake ratio of alanine—C to —N of 0.32:1 indicates that some alanine is likely taken up intact by the plant. Urea—C uptake did not follow the same linear trend as was exhibited by the other measured chemical species. Over 180 minutes, no correlation was found between urea—C uptake and urea—N uptake indicating that urea undergoes rapid extracellular ammonification. Total and speciated N uptake were not statistically different between the three *G. hirsutum* varieties.

6. Acknowledgements

The authors gratefully acknowledge the Cotton Research and Development Corporation (Grant number CSP1904 awarded to BM and MF) for funding, and technical assistance from the ANU Research School of Biology IRMS facility for sample analysis.

7. References

- 1. Jans Y, Von Bloh W, Schaphoff S, Müller C. Global cotton production under climate change – Implications for yield and water consumption. Hydrol Earth Syst Sci. 2021;25(4):2027-44.
- 2. Heffer P, Prud'homme M. Global nitrogen fertiliser demand and supply: trend, current level and outlook. Proceedings of the 2016 International Nitrogen Initiative Conference, "Solutions to improve nitrogen use efficiency for the world", 4 – 8 December 2016, Melbourne, Australia wwwini2016com2016.

- 3. Khan MA, Wahid A, Ahmad M, Tahir MT, Ahmed M, Ahmad S, et al. World Cotton Production and Consumption: An Overview. In: Ahmad S, Hasanuzzaman M, editors. Cotton Production and Uses: Agronomy, Crop Protection, and Postharvest Technologies. Singapore: Springer Singapore; 2020. p. 1-7.
- 4. Macdonald BT, Latimer JO, Schwenke GD, Nachimuthu G, Baird JC. The current status of nitrogen fertiliser use efficiency and future research directions for the Australian cotton industry. JOURNAL OF COTTON RESEARCH. 2018;1.
- 5. Antille DL, Moody PW. Nitrogen use efficiency indicators for the Australian grains, cotton, sugar, dairy, and horticulture industries. Environmental and Sustainability Indicators. 2021;10:Article number: 100099. https://doi.org/10.1016/j.indic.2020..
- 6. Zhang Z, Huang J, Yao Y, Peters G, Macdonald B, La Rosa AD, et al. Environmental impacts of cotton and opportunities for improvement. Nature Reviews Earth & Environment. 2023.
- 7. Macdonald BCT, Nachimuthu G, Chang YF, Nadelko AJ, Tuomi S, Watkins M. Nitrogen composition in furrow irrigated run-off water. Agricultural Water Management. 2020;242:106399.
- 8. Macdonald BCT, Ringrose-Voase AJ, Nadelko AJ, Farrell M, Tuomi S, Nachimuthu G. Dissolved organic nitrogen contributes significantly to leaching from furrow-irrigated cotton-wheat-maize rotations. Soil Research. 2017;55(1):70-7.
- 9. Harris SJ, Cendón DI, Hankin SI, Peterson MA, Xiao S, Kelly BFJ. Isotopic evidence for nitrate sources and controls on denitrification in groundwater beneath an irrigated agricultural district. Science of The Total Environment. 2022;817:152606.
- 10. Grace P, Shcherbak I, Macdonald B, Scheer C, Rowlings D. Emission factors for estimating fertiliser-induced nitrous oxide emissions from clay soils in Australia's irrigated cotton industry. Soil Research. 2016;54(5):598.
- 11. Brackin R, Buckley S, Pirie R, Visser F. Predicting nitrogen mineralisation in Australian irrigated cotton cropping systems. Soil Research. 2019;57(3):247-56.
- 12. Lehmann J, Kleber M. The contentious nature of soil organic matter. Nature. 2015;528(7580):60-8.
- 13. Bond-Lamberty B, Christianson DS, Malhotra A, Pennington SC, Sihi D, AghaKouchak A, et al. COSORE: A community database for continuous soil respiration and other soil-atmosphere greenhouse gas flux data. Glob Chang Biol. 2020.
- 14. Paustian K, Six J, Elliott ET, Hunt HW. Management options for reducing CO2 emission from agricultural soils. Biogeochemistry. 2000;48:147-63.
- 15. Farrell M, Hill PW, Farrar J, Bardgett RD, Jones DL. Seasonal variation in soluble soil carbon and nitrogen across a grassland productivity gradient. Soil Biology and Biochemistry. 2011;43(4):835-44.
- 16. Schimel JP, Bennett J. Nitrogen mineralization: challenges of a changing paradigm. Ecology. 2004;85(3):591-602.
- 17. Virtanen AI, Linkola H. Organic Nitrogen Compounds a Nitrogen Nutrition for Higher Plants. Nature. 1946;158:515.
- 18. Vantsis JT, Bond G. Nitrogen Nutrition of Clover. Nature. 1951;168:339-40.
- 19. Waksman SA. Principles of Soil Microbiology, second edition, thoroughly revised. 2nd ed. Baltimore, Maryland, USA: Williams and Wilkins; 1932.
- 20. Kielland K. Amino Acid Absorption by Arctic Plants: Implications for Plant Nutrition and Nitrogen Cycling. Ecology. 1994;75(8):2373-83.
- 21. Näsholm T, Huss-Danell K, Högberg P. Uptake of organic nitrogen in the field by four agriculturally important plant species. Ecology. 2000;81(4):1155-61.
- 22. Näsholm T, Ekblad A, Nordin A, Giesler R, Högberg M, Högberg P. Boreal forest plants take up organic nitrogen. Nature. 1998;392:914-6.
- 23. Hill PW, Jones DL. Plant-microbe competition: does injection of isotopes of C and N into the rhizosphere effectively characterise plant use of soil N? New Phytol. 2018.
- 24. R Core Team. R: A language and environment for statistical computing. Vienna, Austria: R Foundation for statistical computing; 2020.

- 25. Bates D, Mächler M, Bolker B, Walker S. Fitting Linear Mixed-Effects Models Using lme4. Journal of Statistical Software. 2015;67(1):1 - 48.
- 26. Macdonald BCT, Chang YF, Nadelko A, Tuomi S, Glover M. Tracking fertiliser and soil nitrogen in irrigated cotton: uptake, losses and the soil N stock. Soil Research. 2017;55(3).
- 27. Prendergast-Miller MT, de Menezes AB, Farrell M, Macdonald LM, Richardson AE, Bissett A, et al. Soil nitrogen pools and turnover in native woodland and managed pasture soils. Soil Biology and Biochemistry. 2015;85:63-71.
- 28. Robinson N, Brackin R, Vinall K, Soper F, Holst J, Gamage H, et al. Nitrate paradigm does not hold up for sugarcane. PLoS One. 2011;6(4):e19045.
- 29. Vinall K, Schmidt S, Brackin R, Lakshmanan P, Robinson N. Amino acids are a nitrogen source for sugarcane. Funct Plant Biol. 2012;39(6):503-11.
- 30. Chapin III FS, Moilanen L, Kielland K. Preferential use of organic nitrogen for growth by a non-mychrorrhizal arctic sedge. Nature. 1993;361:150-3.
- 31. Adamczyk B, Godlewski M, Zimny J, Zimny A. Wheat (Triticum aestivum) seedlings secrete proteases from the roots and, after protein addition, grow well on medium without inorganic nitrogen. Plant Biol (Stuttg). 2008;10(6):718-24.
- 32. Liu M, Li C, Xu X, Wanek W, Jiang N, Wang H, et al. Organic and inorganic nitrogen uptake by 21 dominant tree species in temperate and tropical forests. Tree Physiol. 2017;37(11):1515-26.
- 33. Liu M, Xu F, Xu X, Wanek W, Yang X. Age alters uptake pattern of organic and inorganic nitrogen by rubber trees. Tree Physiol. 2018;38(11):1685-93.
- 34. Li C, Li Q, Qiao N, Xu X, Li Q, Wang H. Inorganic and organic nitrogen uptake by nine dominant subtropical tree species. iForest - Biogeosciences and Forestry. 2016;9(2):253-8.
- 35. Leberecht M, Tu J, Polle A. Acid and calcareous soils affect nitrogen nutrition and organic nitrogen uptake by beech seedlings (Fagus sylvatica L.) under drought, and their ectomycorrhizal community structure. Plant and Soil. 2016;409(1-2):143-57.
- 36. Wei L, Chen C, Yu S. Uptake of organic nitrogen and preference for inorganic nitrogen by two Australian native Araucariaceae species. Plant Ecology & Diversity. 2014;8(2):259-64.
- 37. Gruffman L, Jamtgard S, Nasholm T. Plant nitrogen status and co-occurrence of organic and inorganic nitrogen sources influence root uptake by Scots pine seedlings. Tree Physiol. 2014;34(2):205-13.
- 38. Wei L, Chen C, Xu Z, Näsholm T. Direct uptake and rapid decrease of organic nitrogen by Wollemia nobilis. Biol Fertil Soils. 2013;49(8):1247-52.
- 39. Gruffman L, Palmroth S, Nasholm T. Organic nitrogen uptake of Scots pine seedlings is independent of current carbohydrate supply. Tree Physiol. 2013;33(6):590-600.
- 40. Jiang L, Wang S, Pang Z, Wang C, Kardol P, Zhou X, et al. Grazing modifies inorganic and organic nitrogen uptake by coexisting plant species in alpine grassland. Biol Fertil Soils. 2015;52(2):211-21.
- 41. Moore TR, Alfonso A, Clarkson BR. Plant uptake of organic nitrogen in two peatlands. Plant and Soil. 2018;433(1-2):391-400.
- 42. Xu X, Ouyang H, Cao G, Pei Z, Zhou C. Uptake of organic nitrogen by eight dominant plant species in Kobresia meadows. Nutrient Cycling in Agroecosystems. 2004;69:5-10.
- 43. Dahlman L, Persson J, Palmqvist K, Nasholm T. Organic and inorganic nitrogen uptake in lichens. Planta. 2004;219(3):459-67.
- 44. Song L, Lu HZ, Xu XL, Li S, Shi XM, Chen X, et al. Organic nitrogen uptake is a significant contributor to nitrogen economy of subtropical epiphytic bryophytes. Sci Rep. 2016;6:30408.
- 45. Dion PP, Jamtgard S, Bertrand A, Pepin S, Dorais M. Organic Nitrogen Uptake and Assimilation in Cucumis sativus Using Position-Specific Labeling and Compound-Specific Isotope Analysis. Front Plant Sci. 2018;9:1596.
- 46. Zhou B, Zhang L, Yang W, Mao Y, Chen C, Xing S. Differential uptake of soluble organic and inorganic nitrogen by two fruit species: Dimocarpus longan Lour. and Eriobotrya japonica Lindl. J Soils Sed. 2016;17(6):1579-87.

- 47. Moran-Zuloaga D, Dippold M, Glaser B, Kuzyakov Y. Organic nitrogen uptake by plants: reevaluation by position-specific labeling of amino acids. Biogeochemistry. 2015;125(3):359-74.
- 48. Saia S, BenÍTez E, GarcÍA-Garrido JM, Settanni L, Amato G, Giambalvo D. The effect of arbuscular mycorrhizal fungi on total plant nitrogen uptake and nitrogen recovery from soil organic material. The Journal of Agricultural Science. 2013;152(03):370-8.
- 49. Näsholm T, Huss-Danell K, Högberg P. Uptake of glycine by field grown wheat. New Phytol. 2000;150:59-63.
- 50. Gallet-Budynek A, Brzostek E, Rodgers VL, Talbot JM, Hyzy S, Finzi AC. Intact amino acid uptake by northern hardwood and conifer trees. Oecologia. 2009;160(1):129-38.
- 51. Farrell M, Prendergast-Miller M, Jones DL, Hill PW, Condron LM. Soil microbial organic nitrogen uptake is regulated by carbon availability. Soil Biology and Biochemistry. 2014;77:261-7.
- 52. Hill PW, Marsden KA, Jones DL. How significant to plant N nutrition is the direct consumption of soil microbes by roots? New Phytol. 2013;199(4):948-55.
- 53. Paungfoo-Lonhienne C, Rentsch D, Robatzek S, Webb RI, Sagulenko E, Nasholm T, et al. Turning the table: plants consume microbes as a source of nutrients. PLoS One. 2010;5(7):e11915.
- 54. Perchlik M, Tegeder M. Leaf Amino Acid Supply Affects Photosynthetic and Plant Nitrogen Use Efficiency under Nitrogen Stress. Plant Physiol. 2018;178(1):174-88.
- 55. Feller U, Anders I, Mae T. Rubiscolytics: fate of Rubisco after its enzymatic function in a cell is terminated. J Exp Bot. 2008;59(7):1615-24.

Table 1 Nutrient solution prepared based on the Hoaglands solution and recommendations from Oliver Knox, University of New England. Mass and mol per cent are calculated excluding oxygen and hydrogen. Solution was pH balanced to 7 ± 0.05 and EC kept below 1000 µS cm^{-1} to reduce the chance of acidification or salinisation.

Element	Mass Per Cent	Mol Per Cent	Compounds
N	25.17%	44.29%	KNO_3 , $Ca(NO_3)_2$, FeNaEDTA
P	3.61%	2.88%	KH_2PO_4
K	27.38%	17.25%	KNO_3
Ca	23.38%	14.38%	$Ca(NO_3)_2$
C	2.80%	5.75%	FeNaEDTA
S	7.48%	5.75%	$MgSO_4$
Mg	5.67%	5.75%	$MgSO_4$
Na	1.08%	1.16%	FeNaEDTA , $Na_2MoO_4{\cdot}2H_2O$
Cl	0.85%	0.59%	$MnCl_2{\cdot}4H_2O$, $ZnCl_2$, $CuCl_2{\cdot}2H_2O$
B	0.584%	1.330%	H_3BO_3
Fe	1.303%	0.575%	FeNaEDTA
Mn	0.586%	0.263%	$MnCl_2{\cdot}4H_2O$
Zn	0.062%	0.023%	$ZnCl_2$
Cu	0.022%	0.008%	$CuCl_2{\cdot}2H_2O$
Mo	0.012%	0.003%	$Na_2MoO_4{\cdot}2H_2O$

Table 2 Nitrogen treatments for each of the three G. hirsutum *varieties included in this study. The total N concentration of all solutions was 2.95 mmol N L^{-1}, with approximately 1 mmol N L^{-1} apportioned to each of the three plant-available N pools outlined in this study: mineral N, synthetic N and DON.*

	**N Treatment**	**Nitrate**	**Ammonium**	**Urea**	**Alanine**
**Plant-Available N Pool:**		Mineral N	Mineral N	Synthetic N	LMW DON
**Nitrogen Isotope Purity:**		99.8% ^{15}N	99.5% ^{15}N	99% ^{15}N	99% ^{15}N
**Carbon Isotope Purity:**		-	-	98% ^{13}C	99% ^{13}C
**Concentration (mmol N L^{-1}):**		0.61	0.35	1.00	1.00
**Concentration (mmol C L^{-1}):**		-	-	0.50	3.00
	0	Unlabelled	Unlabelled	Unlabelled	Unlabelled
	1	^{15}N-Labelled	Unlabelled	Unlabelled	Unlabelled
	2	Unlabelled	^{15}N-Labelled	Unlabelled	Unlabelled
	3	Unlabelled	Unlabelled	^{15}N-^{13}C-Labelled	Unlabelled
	4	Unlabelled	Unlabelled	Unlabelled	^{15}N-^{13}C-Labelled

Table 3. G. hirsutum *estimated marginal mean uptake of N species after 180 minutes of exposure. Values are mean ± standard error, n = 10.*

**Nitrogen Species**	**% uptake of the added N**
Alanine	14.3 ± 1.9%
Ammonium	16.6 ± 1.9%
Nitrate	25.1 ± 1.9%
Urea	22.2 ± 1.9%
Inorganic N (NO_3^- + NH_4^+)	20.9 ± 3.8%
Organic N (alanine + urea)	18.3 ± 0.38%

Table 4. *Linear N uptake rate of each fertiliser species for average of all three* G. hirsutum *varieties (n = 90).*

**Nitrogen Species**	**Uptake Rate** (% uptake per hour)	**Uptake Rate** (Hours for 100% Uptake)	**Fit (r^2)**
Alanine	4.6%	21.7	0.997
Ammonium	5.4%	18.6	0.997
Nitrate	8.1%	12.3	0.994
Urea	7.1%	14.1	0.973

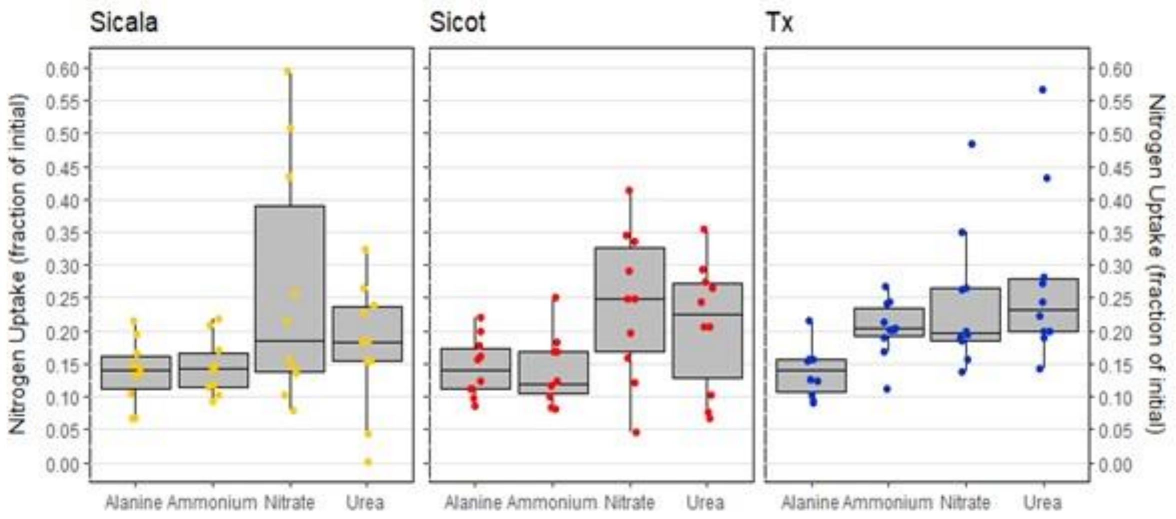

Figure 1. N uptake after 180 minutes (nominal) for each G. hirsutum variety: Sicot 746B3F, Sicala V2 and Tx III. The four N compounds NO_3^-, NH_4^+, urea (CH_4N_2O) and alanine ($C_3H_7NO_2$) were added concurrently.

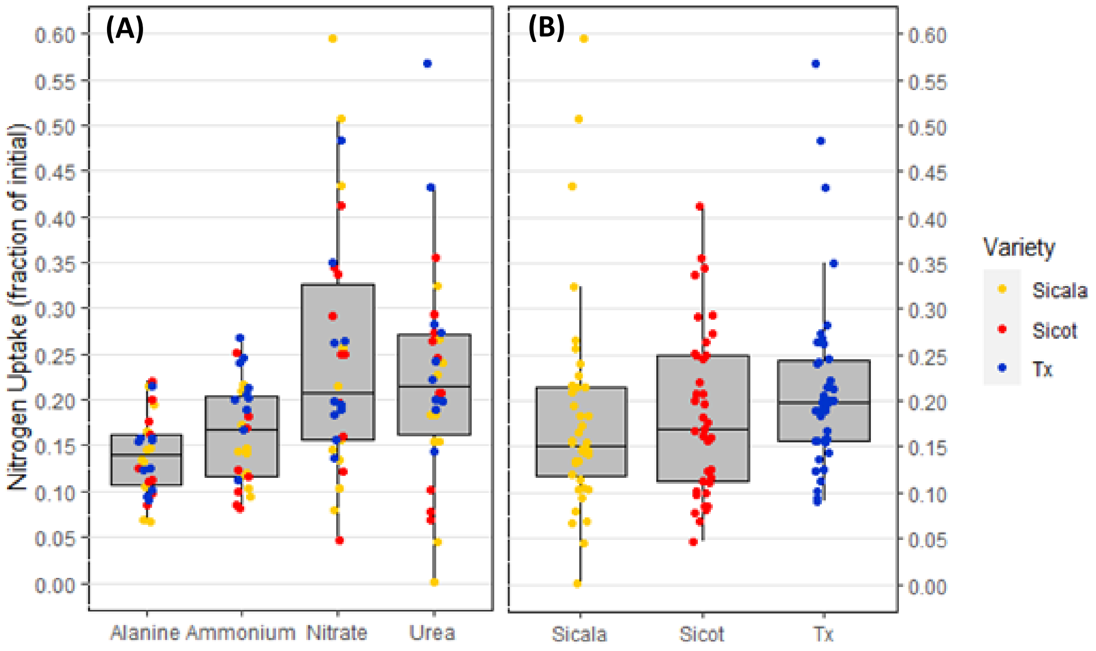

Figure 2. Nitrogen uptake after 180 minutes (nominal). **(A) A**verage N compound uptake for all varieties. **(B)** Total N uptake for each G. hirsutum variety.

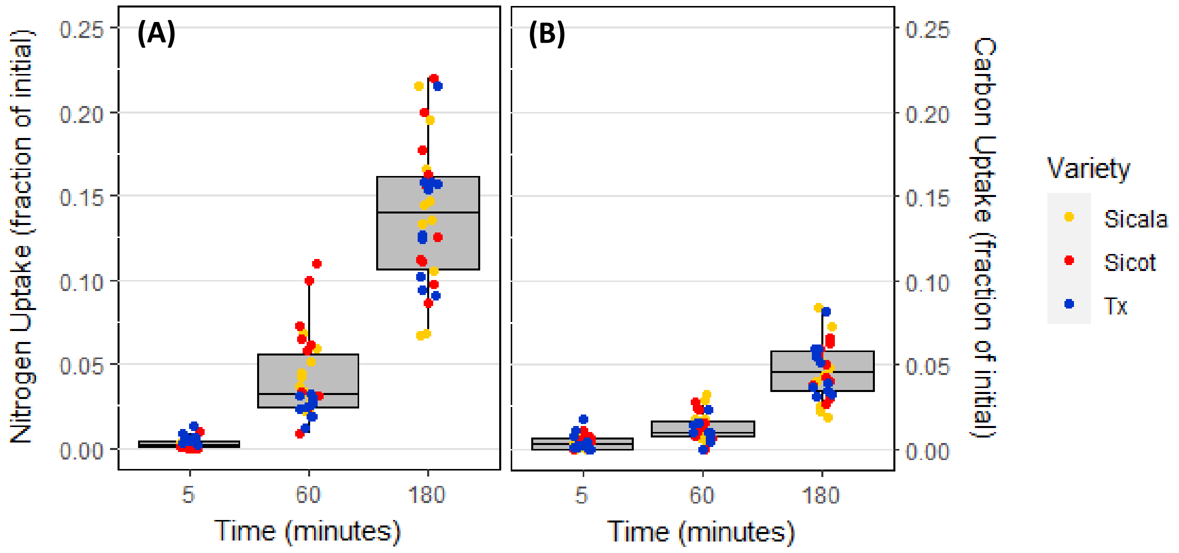

***Figure 3**. Average alanine uptake of all* G. hirsutum *varieties within the three sampling intervals.*

***(A)** Alanine—N uptake. **(B)** Alanine—C uptake.*

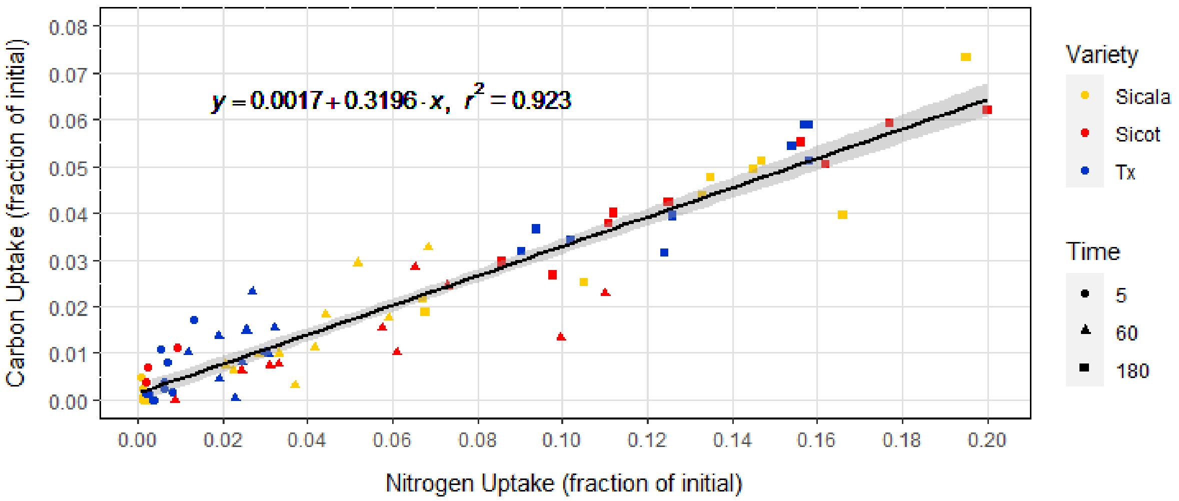

Figure 4. *Alanine—C uptake against alanine—N uptake plotted as fraction of initial concentration. Equation describes the linear regression that is plotted in black with 95% confidence interval in grey. Data point shapes denote isotope exposure times in minutes. The uptake of alanine—C and alanine—N are highly correlated ($r^2 = 0.92$, $p < 0.001$).*

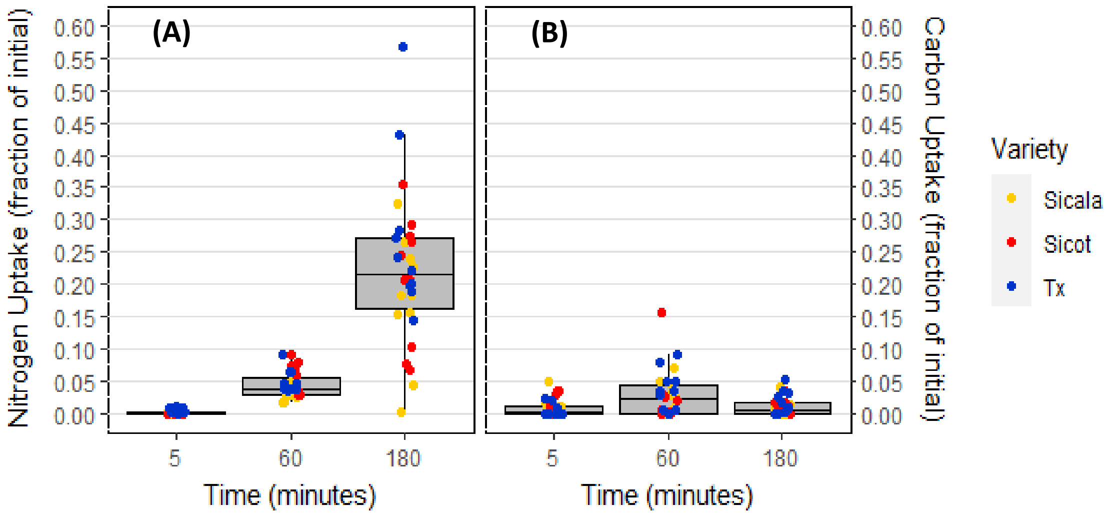

***Figure 5** Average urea uptake of all* G. hirsutum *varieties over the three sampling intervals. **(A)** Urea—N uptake. **(B)** Urea—C uptake, which does not increase consistently over time.*

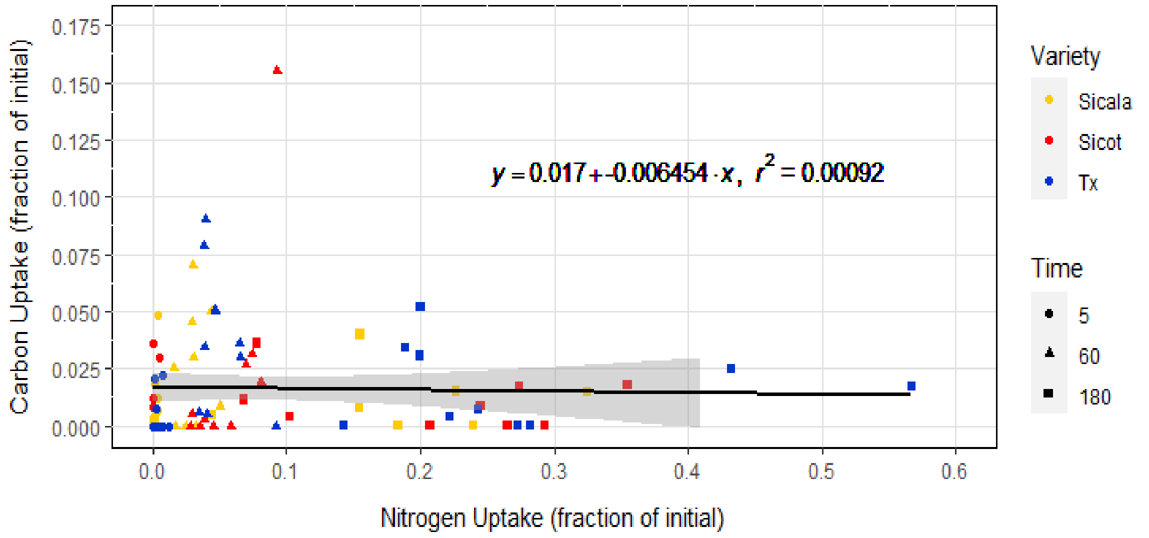

Figure 6. *Urea—C uptake against urea—N uptake plotted as fraction of initial concentration. Equation describes the linear regression that is plotted in black with 95% confidence interval in grey. Data point shapes denote isotope exposure times in minutes. Urea-N and urea-C uptake do not correlate over the whole 180 minute experimental window (*$r^2 < 0.001$*, p = 0.96).*

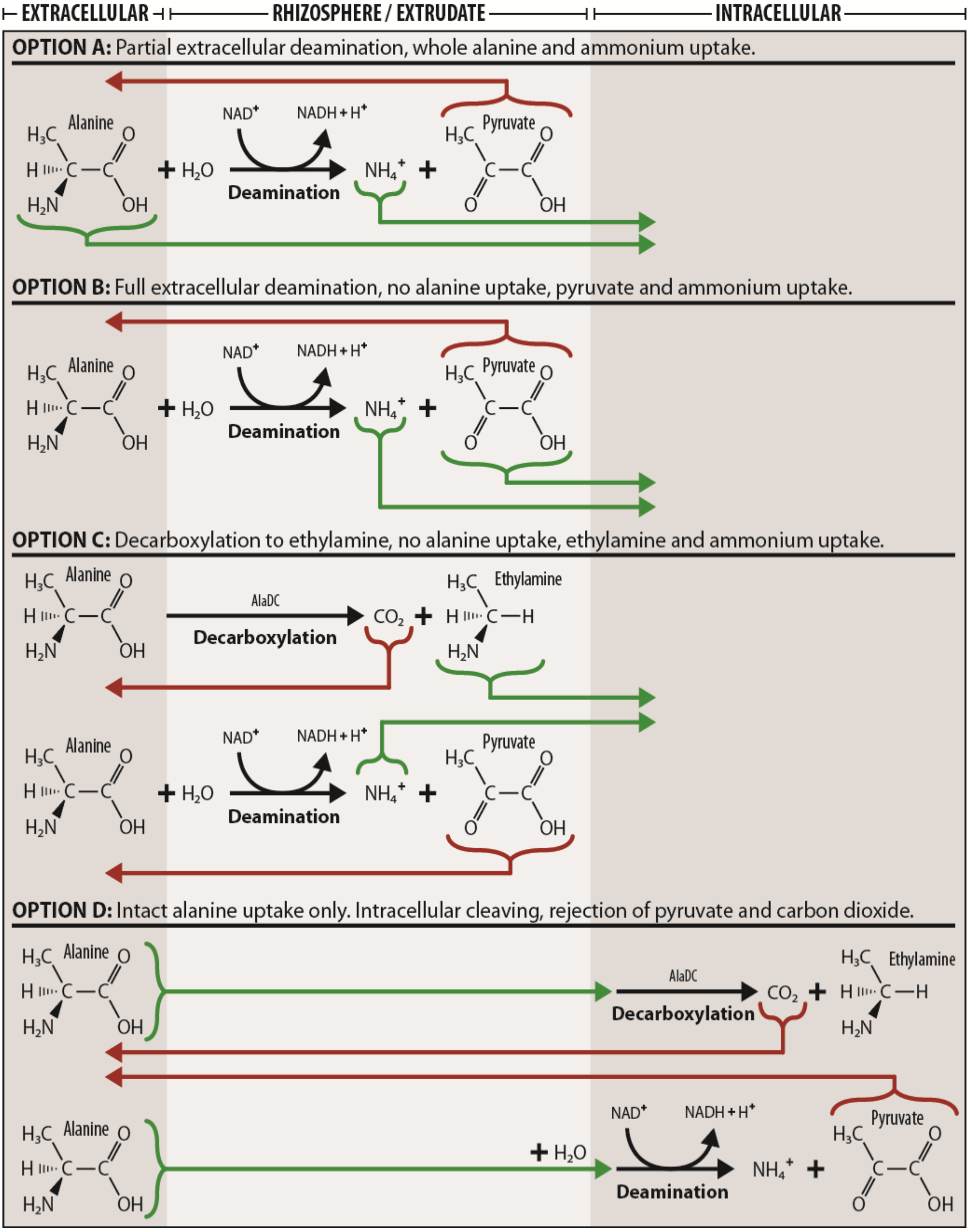

***Figure 7**. Four potential alanine—N and –C uptake scenarios. Experimental results suggest the majority of alanine—N is taken up as an amine functional group, potentially NH_4^+, which would likely mean extracellular deamination. Alanine—C uptake suggests that up to 32% of alanine—N was taken up as whole alanine molecules. Actual uptake behaviour may be a combination, or none, of these options. AlaDC is an abbreviation of the enzyme alanine decarboxylase.*

438

Supplement Figures

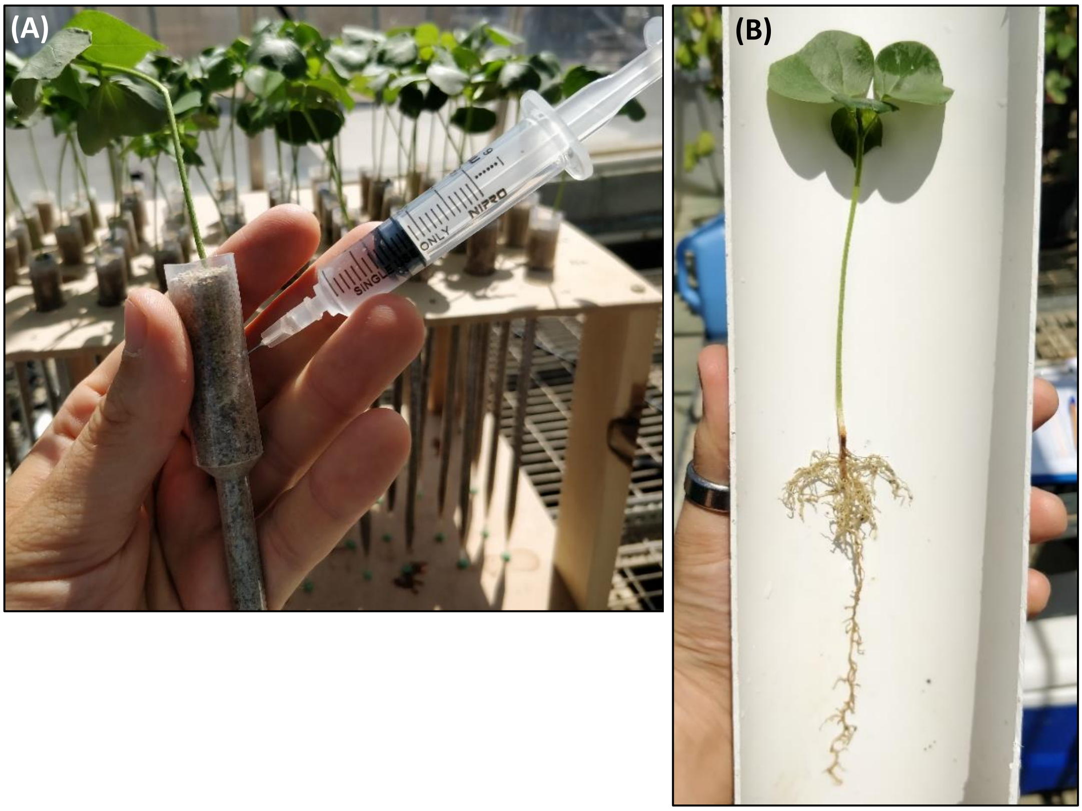

Figure S1. Processing of *G. hirsutum* seedlings in $^{15}N^{13}C$ uptake glasshouse experiment. **(A)** Injection of rhizotube with N solution. Careful attention was paid to not inject solution directly into the plant. **(B)** Extracted and cleaned *G. hirsutum* seedling. Once cleaned, seedlings were bagged and placed on dry ice ($CO_{2(s)}$) to halt metabolism.

Figure S2

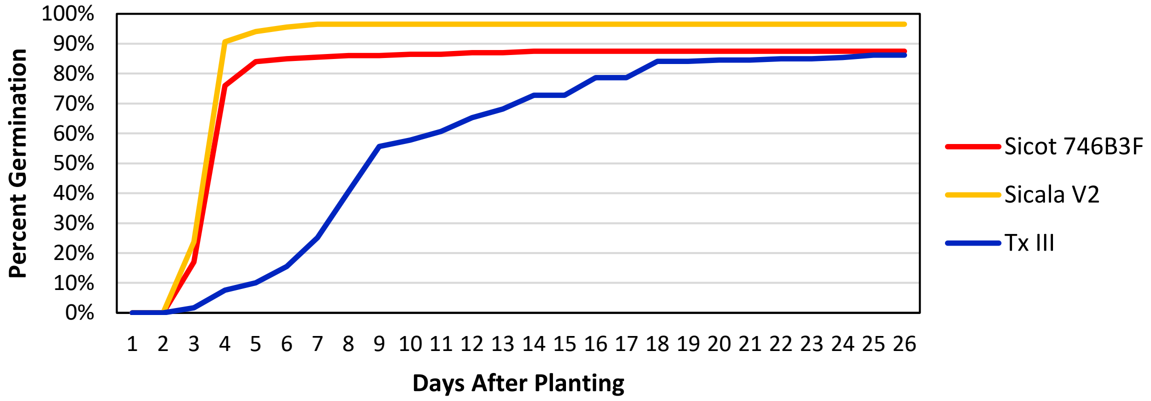

Figure S2. *Germination percentage for each of the three* G. hirsutum *varieties: Sicot 746B3F, a GM current commercial cultivar; Sicala V2, an obsolete non-GM commercial cultivar; and Tx III, a Guatemalan landrace accession. n = 720 seeds.*
