# Supplementary material for: Nitrogen uptake preference of cotton (Gossypium hirsutum L.)
Source: PLoS One. 2025 Dec 12;20(12):e0334700. doi: 10.1371/journal.pone.0334700 (PMC12700366; doi:10.1371/journal.pone.0334700)
Supplement: Attachment — Submitted filename: Review report.docx [file pone.0334700.s003.docx]

The manuscript entitled “Nitrogen uptake preference of Gossypium hirsutum L. from a mixture of urea, alanine, nitrate, and ammonium" has been reviewed. The authors provided the information on the ability of G. hirstutum to take up different organic and inorganic N forms in a sand matrix. It is well known that plants always take nitrogen in inorganic form. The manuscript, at least as written, lacks novelty. Despite the fact that, I provided some points to the authors which are address below. I have also highlighted and commented some points in Manuscript.

- The English in the paper is very rough and requires the assistance of a more practiced English expert to make the paper more easily read and understandable.
- The abstract still requires substantial editing.
- The Purpose of the study in the abstract section is not clear. It should be concise within two to three lines.
- Conclusion part in abstract section needs to be rewrite, it should be clear for better understanding of the readers
- The quality of writing should be improved.
- Introduction section is deficient. The authors did not present a novel justification for carrying out this study.
- There is a dire need to address grammatical errors, as the sentence structure is unclear and confusing. This will make it difficult for readers to grasp the intended meaning.
- The results and discussion section should be improved, well-structured and interconnected and the discussion should be link to all your findings.
- The authors should finish the discussion with what are the main points (take home massage).
- The paragraphs in result discussion section seems to be correspond to the results only and not to a real discussion of your results. The paragraphs are not appropriately discussed.
- The deeper scientific interpretations of your findings are strongly suggested.
- The conclusion must be self-explanatory and highlight novelty and implication of your study.
- Make sure that all figures and tables are cited within the text and that they are cited in consecutive order.
- References should be in Format
- Please use Some recent references
