# Supplementary material for: Nitrogen uptake preference of cotton (Gossypium hirsutum L.)
Source: PLoS One. 2025 Dec 12;20(12):e0334700. doi: 10.1371/journal.pone.0334700 (PMC12700366; doi:10.1371/journal.pone.0334700)
Supplement: Attachment — Submitted filename: Reviewers Comments.docx [file pone.0334700.s005.docx]

**Comments on Manuscript Number: PONE-D-24-59331**

The study aims to decipher N uptake preference from N sources viz. urea, alanine, nitrate, and ammonium through isotopic studies and probable mechanism thereof.

The methodology is robust and all the results pertaining to the objective are elaborated. The paper may be accepted after minor revisions as mentioned below:

Check the subheadings. Some subheadings are in running sentences while in some, each words are capitalized .

Abbreviations used in the paper must be described at the first mention and used uniformly thereafter.

Line number 45: check spelling of minerialisation

Line number 98-100: Mention the statistical design
Line number 111: (s) should be normal font size?

Line number 123, 127….: Equation may be presented a	(1)

Line number 139: R version may be mentioned

In some, species are mentioned as NH_4_^+^–N and alanine—N while in some cases NH_4_^+^, alanine, etc are used. Uniform names may be followed.

References may be double-checked to follow the journal referencing guidelines and follow uniform referencing style throughout. Double-check to make sure that all references have a corresponding citation within the text and vice versa.
