# Supplementary material for: Nitrogen uptake preference of cotton (Gossypium hirsutum L.)
Source: PLoS One. 2025 Dec 12;20(12):e0334700. doi: 10.1371/journal.pone.0334700 (PMC12700366; doi:10.1371/journal.pone.0334700)
Supplement: Attachment — Submitted filename: Response to reviewers.docx [file pone.0334700.s008.docx]

**Response to reviewers – PONE-D-24-59331**

We thank the reviewers and editorial time in handling and reviewing our submission to PLoS1. We are pleased to address these comments, and our responses are given below in blue. As requested, we have also submitted a version of the MS with changes tracked to better facilitate the review process. All line numbers in our responses refer to the line numbers in the revised, clean version.

*Editorial comments:*

Journal Requirements:

When submitting your revision, we need you to address these additional requirements.

1.Please ensure that your manuscript meets PLOS ONE's style requirements, including those for file naming. The PLOS ONE style templates can be found at

<https://journals.plos.org/plosone/s/file?id=wjVg/PLOSOne_formatting_sample_main_body.pdf> and

[https://journals.plos.org/plosone/s/file?id=ba62/PLOSOne_formatting_sample_title_authors_affiliations.pdf](https://track.editorialmanager.com/CL0/https:%2F%2Fjournals.plos.org%2Fplosone%2Fs%2Ffile%3Fid=ba62%2FPLOSOne_formatting_sample_title_authors_affiliations.pdf/1/010f01959488000a-11dfa762-2af1-40c0-941c-c6a4ca949953-000000/IOhcGhQKOUSsYBHUMQwhxbTj-H7MjTYSd0qVnOSP1zI=202)

Thank you for highlighting these issues. We apologies for not having thoroughly attended to these issues prior to the first submission, and hope that they are now deemed addressed satisfactorily. We will be happy to make any further modifications as required to meet the Journal’s specifications.

2. Please note that funding information should not appear in any section or other areas of your manuscript. We will only publish funding information present in the Funding Statement section of the online submission form. Please remove any funding-related text from the manuscript.

This is now done

3. When completing the data availability statement of the submission form, you indicated that you will make your data available on acceptance. We strongly recommend all authors decide on a data sharing plan before acceptance, as the process can be lengthy and hold up publication timelines. Please note that, though access restrictions are acceptable now, your entire data will need to be made freely accessible if your manuscript is accepted for publication. This policy applies to all data except where public deposition would breach compliance with the protocol approved by your research ethics board. If you are unable to adhere to our open data policy, please kindly revise your statement to explain your reasoning and we will seek the editor's input on an exemption. Please be assured that, once you have provided your new statement, the assessment of your exemption will not hold up the peer review process.

All authors have agreed to this statement. Publication of data relating to publications on the CSIRO Data Access Portal (data.csiro.au) is routine for publications arising from CSIRO authors.

4. PLOS requires an ORCID iD for the corresponding author in Editorial Manager on papers submitted after December 6th, 2016. Please ensure that you have an ORCID iD and that it is validated in Editorial Manager. To do this, go to ‘Update my Information’ (in the upper left-hand corner of the main menu), and click on the Fetch/Validate link next to the ORCID field. This will take you to the ORCID site and allow you to create a new iD or authenticate a pre-existing iD in Editorial Manager.

Apologies. This has now been completed by all authors.

5. We note you have included a table to which you do not refer in the text of your manuscript. Please ensure that you refer to Table 4 in your text; if accepted, production will need this reference to link the reader to the Table.

Apologies, this is now referenced at L169

Additional Editor Comments:

The authors are advised to revise the manuscript according to the reviewers' comments and resubmit it for further review.

*Reviewer 1:*

The manuscript entitled “Nitrogen uptake preference of Gossypium hirsutum L. from a mixture of urea, alanine, nitrate, and ammonium" has been reviewed. The authors provided the information on the ability of G. hirstutum to take up different organic and inorganic N forms in a sand matrix. It is well known that plants always take nitrogen in inorganic form. The manuscript, at least as written, lacks novelty. Despite the fact that, I provided some points to the authors which are address below. I have also highlighted and commented some points in Manuscript.

We thank the reviewer for their comments. We contest their statement that “it is well known that plants always take nitrogen in inorganic form” as this is an inaccurate viewpoint that has been superseded over the past three decades of research into organic nitrogen nutrition of many species of higher plants (e.g., grasses, forbs, shrubs, trees, cereals), demonstrating their capacity to access organic nitrogen in the form of amino acids, short peptides, quaternary ammonium compounds, and even potentially whole proteins and microorganisms. Our data clearly demonstrate for the first time that the economically important *G. hirsutum* can now be added to that list.

- The English in the paper is very rough and requires the assistance of a more practiced English expert to make the paper more easily read and understandable.

All three authors are native English speakers. In redrafting this revision, we have made adjustments where extra clarity was required, and have also addressed the specific points raised by this reviewer.

- The abstract still requires substantial editing.

We have revised the abstract in line with the specific comments below from this reviewer

- The Purpose of the study in the abstract section is not clear. It should be concise within two to three lines.

We have clearly stated the purpose in response to this request

- Conclusion part in abstract section needs to be rewrite, it should be clear for better understanding of the readers

We have now re-arranged and expanded the concluding remarks of the abstract

- The quality of writing should be improved.

Please see earlier response

- Introduction section is deficient. The authors did not present a novel justification for carrying out this study.

We emphasise that the ability of the commercially important *G. hirsutum* to access organic N has not been previously examined at L75.

- There is a dire need to address grammatical errors, as the sentence structure is unclear and confusing. This will make it difficult for readers to grasp the intended meaning.

We have addressed the specific points on clarity raised by this reviewer below.

- The results and discussion section should be improved, well-structured and interconnected and the discussion should be link to all your findings.

Separating the results and discussion sections is preferred as this allows clear distinctions to be drawn between the findings of the study (results section) and their interpretation in light of published studies (discussion section). Specific comments raised by this reviewer have been addressed below.

- The authors should finish the discussion with what are the main points (take home massage).

The discussion is structured into sub-sections, as is common in scientific writing. We have taken care to ensure that each subsection now clearly emphasises its take-home message.

- The paragraphs in result discussion section seems to be correspond to the results only and not to a real discussion of your results. The paragraphs are not appropriately discussed.

As per our earlier response, the purpose of separating the results and discussion sections enables a delineation between the findings of the study (results section) and its implications in the context of the wider literature (discussion section).

- The deeper scientific interpretations of your findings are strongly suggested.

This study targeted examination of the physiological capacity of *G. hirsutum* to take up organic N forms, rather than questioning whether it does so in competition with soil microorganisms in field conditions. We highlight this limitation at L197. Thus, whilst we have modified text throughout the discussion, we do not feel it appropriate to over-interpret these data.

- The conclusion must be self-explanatory and highlight novelty and implication of your study.

We agree that the conclusion was not satisfactory and have completely revised this section.

- Make sure that all figures and tables are cited within the text and that they are cited in consecutive order.

This has now been checked

- References should be in Format

We have used the PLoS format in EndNote, and this appears to match that of the PLoS One guidelines

- Please use Some recent references

Two recent references (Gao et al 2025 #23, Bailey et al 2022 #51) have been added

L1	Improve the title

We have now simplified the title

L19	Rewrite the sentence. Not clear

Now done

L37	Sentenceis not clear. Not understanding the meaning of sentence.

Now revised for clarity

L59	Very big sentence, not clear to understand, some words are missing, role of microbes. please try to read and rewrite again.

Now revised for clarity

L126	Write in seperate line for more clear picture and understandin

Now revised for clarity

L135	two times solution please write the sentence clearly

Now revised for clarity

L136	Superscript

Thank you - fixed

L241	Check and rewrite

Sentence has been removed as part of the broader re-write and improvements of the conclusions section

*Reviewer 2:*

The study aims to decipher N uptake preference from N sources viz. urea, alanine, nitrate, and ammonium through isotopic studies and probable mechanism thereof.

The methodology is robust and all the results pertaining to the objective are elaborated. The paper may be accepted after minor revisions as mentioned below:

We thank the reviewer for their supportive and constructive comments

Check the subheadings. Some subheadings are in running sentences while in some, each words are capitalized .

This has now been resolved

Abbreviations used in the paper must be described at the first mention and used uniformly thereafter.

This has now been checked and resolved. The only exception to this is in discussion of the results where we do use NH4_+_^-^-N and NO_3_^-^-N to be accurate in the comparison with the N from the organic N cpds (e.g. alanine-N) as of course we only track the 15N and not the entire molecule. Thus we are being specific in talking about the N (or C) from each source molecule.

Line number 45: check spelling of minerialisation

Fixed, thank you

Line number 98-100: Mention the statistical design

Now done, thank you

Line number 111: (s) should be normal font size?

Fixed, thank you

Line number 123, 127….: Equation may be presented a	(1)

Now done, thank you

Line number 139: R version may be mentioned

Now done, thank you

In some, species are mentioned as NH_4_^+^–N and alanine—N while in some cases NH_4_^+^, alanine, etc are used. Uniform names may be followed.

We explicitly highlight the –N in these cases when discussing the results as the isotopic analysis only allows us to be absolutely certain about the N (or C) and not the rest of the molecule in question. So, to treat N from nitrate or N from alanine equally, we must emphasise that it is only the N that is being discussed and not the rest of the molecule (that can only be presumed to have followed).

References may be double-checked to follow the journal referencing guidelines and follow uniform referencing style throughout. Double-check to make sure that all references have a corresponding citation within the text and vice versa.

This has now been done. We used the Endnote style for PLOS and made minor modifications to this to match current formatting instructions on the PLOS1 website.
