# Supplementary material for: Nitrogen uptake preference of cotton (Gossypium hirsutum L.)
Source: PLoS One. 2025 Dec 12;20(12):e0334700. doi: 10.1371/journal.pone.0334700 (PMC12700366; doi:10.1371/journal.pone.0334700)
Supplement: Attachment — Submitted filename: PONE-D-24-59331.docx [file pone.0334700.s007.docx]

Line no. 2: Is BCT the abbreviation for Bennet C.T.? If yes, is it suitable to remove BCT?

Line no. 21: Two full stops, remove one of them

Line no. 28: What do you mean by taking up intact? Do you mean to say that they are taken up in compound form without any dissociation?

Line no. 72: DON?? Abbreviate it first

Line no. 74: inconcert or in concert?

Line no. 41: compare the dash used in line 41, 46,142, 169 similar dashes may be used

Line no. 117: give space between 40 and °C

Line no. 193: check spelling of demomnstrate

Line no. 196: soil N pool or soil organic n pool?

Line no. 264: add space between G. hirsutum and through

Line no. 271: correct the spelling of biogeochmaical

In references, the number is repeated. Remove one of them. The format needs to be revised, for example, some of the journal name are in abbreviation while some are in full, some doi is presented as doi: while some are in https://doi.org/ , see the journal format again.
