# Supplementary material for: Nitrogen uptake preference of cotton (Gossypium hirsutum L.)
Source: PLoS One. 2025 Dec 12;20(12):e0334700. doi: 10.1371/journal.pone.0334700 (PMC12700366; doi:10.1371/journal.pone.0334700)
Supplement: Attachment — Submitted filename: 2025-08 latimeretal_R1 Revisions.docx [file pone.0334700.s009.docx]

Reviewer #2:

- Line no. 2: Is BCT the abbreviation for Bennet C.T.? If yes, is it suitable to remove BCT? **[BCT removed]**
- Line no. 21: Two full stops, remove one of them **[removed]**
- Line no. 28: What do you mean by taking up intact? Do you mean to say that they are taken up in compound form without any dissociation? **[have added “…was absorbed intact without extracellular deamination.”]**
- Line no. 72: DON?? Abbreviate it first **[It was defined on line 58]**
- Line no. 74: inconcert or in concert? **[space inserted]**
- Line no. 41: compare the dash used in line 41, 46,142, 169 similar dashes may be used **[These dashes all converted to standard “-“ dash. All em dashes, en dashes and hyphens have been reviewed in the document**

**Em dash (—)	e.g. alanine—N
En dash (–)	e.g. NH_4_^+^–N
Hyphen (-)	e.g. 40-50**

**]**

- Line no. 117: give space between 40 and °C **[space inserted]**
- Line no. 193: check spelling of demomnstrate **[corrected]**
- Line no. 196: soil N pool or soil organic n pool? **[reworded for clarity]**
- Line no. 264: add space between G. hirsutum and through **[corrected]**
- Line no. 271: correct the spelling of biogeochmaical **[corrected]**

Additional Editor Comments:

- There is no rationale why only alanine and urea as an organic N form is selected as treatment because there are many other organic N forms are available? It is somehow reflected in hypothesis, but a clear statement would help. **[Alanine is a simple model organic N compound that is a major component of proteins – the main non-synthetic source of organic N in soils. Alanine has been used previously as a model organic N in studies investigating both plant (Hill et al. 2011, PLoS1) and microbial (Farrell et al. 2011, GBC) organic N uptake, and has been shown to be intermediate in turnover time relative to a number of other amino acids (Farrell et al. 2014, SBB). Urea was used as the alternate organic N compound as it is a commonly used N fertiliser, especially in irrigated cotton system, where its high N density is beneficial for transport logistics. We have added an explanatory sentence detailing this after the hypotheses.]**
- You have used labelled N and C to exactly quantify the N uptake by cotton, I suggest making a graphical abstract showing how much proportion of N uptake through several N forms. **[completed]**
- L123, How the 13C information used in N uptake (equation 1-4)? **[Line 129 explains that the equations’ pronumerals should be substituted when calculating carbon. Alternatively, we could list each equation twice next to each other with each of the appropriate pronumerals if that is preferred]**
- L188, present r coeff value. Is this a no correlation or non-significant correlation? **[There is no correlation is demonstrated in the figure and thus we do not report a value in text]**
- L264, L266, Fix syntax error (eg space and spelling) **[We believe this is now fixed]**
- This is 180 minutes research suggesting a N uptake preference of cotton. Please justify why only 180 minutes, people working on isotope might understand but it is good to provide some rationale to clarify wider audience. **[Due to the well documented rapid turnover of amino acids and other low molecular weight dissolved organic N compounds in soil, longer term pulse-chase experiments risk only observing uptake dominated by N (and C) from the target compounds after extracellular mineralisation of the target labelled compound. The very short-term nature of this experiment and the sand matrix growth medium were chosen to best probe N uptake dominated by intact compounds rather than the secondary products of extracellular mineralisation. A short sentence explaining this has been added to the end of the Experimental Design sub-section.]**
